# Adaptive evolution in the toxicity of a spider’s venom enzymes

**DOI:** 10.1186/s12862-015-0561-4

**Published:** 2015-12-21

**Authors:** Aurélio Pedroso, Sergio Russo Matioli, Mario Tyago Murakami, Giselle Pidde-Queiroz, Denise V. Tambourgi

**Affiliations:** Laboratório de Imunoquímica, Instituto Butantan, São Paulo, S.P. Brazil; Departamento de Genética e Biologia Evolutiva, Instituto de Biociências, Universidade de São Paulo, São Paulo, S.P. Brazil; Laboratório Nacional de Biociências, Centro Nacional de Pesquisa em Energia e Materiais, Campinas, S.P. Brazil

**Keywords:** Molecular evolution, Phylogenetics, Bioinformatics, Enzyme, Toxin, Immunochemistry, Phospholipase D, Toxicity, Sphingomyelinase D, Spider venom

## Abstract

**Background:**

Sphingomyelinase D is the main toxin present in the venom of *Loxosceles* spiders. Several isoforms present in these venoms can be structurally classified in two groups. Class I Sphingomyelinase D contains a single disulphide bridge and variable loop. Class II Sphingomyelinase D presents an additional intrachain disulphide bridge that links a flexible loop with a catalytic loop. These classes exhibit differences in their toxic potential. In this paper we address the distribution of the structural classes of SMase D within and among species of spiders and also their evolutionary origin by means of phylogenetic analyses. We also conducted tests to assess the action of natural selection in their evolution combined to structural modelling of the affected sites.

**Results:**

The majority of the Class I enzymes belong to the same clade, which indicates a recent evolution from a single common ancestor. Positively selected sites are located on the catalytic interface, which contributes to a distinct surface charge distribution between the classes. Sites that may prevent the formation of an additional bridge were found in Class I enzymes.

**Conclusions:**

The evolution of Sphingomyelinase D has been driven by natural selection toward an increase in noxiousness, and this might help explain the toxic variation between classes.

**Electronic supplementary material:**

The online version of this article (doi:10.1186/s12862-015-0561-4) contains supplementary material, which is available to authorized users.

## Background

Sphingomyelinase D (SMase D; sphingomyelin phosphodiesterase D; E.C. 3.1.4.41) is the main toxin present in the venom of *Loxosceles* spp. spiders and is responsible for the dermonecrosis and systemic effects observed in loxoscelism [1–3]. SMase D is a phospholipase that catalyses the hydrolysis of sphingomyelin (SM), the major constituent in the outer surface of the lipid bilayer of most eukaryotic plasma membranes. The effects of this enzyme result in the formation of ceramide 1-phosphate (*N*-acylsphingosine 1-phosphate) and choline, whereas mammalian sphingomyelinases convert SM in phosphocholine and ceramide [1–5].

*Loxosceles* spiders express several highly homologous isoforms of SMase D with an identity varying from 40 to 90 % [6]; thus, they likely possess the same (α/β)_8_ or TIM barrel fold [7, 8]. A scheme for the classification of SMases D [8] in the spider venom was proposed based on the sequence identity, biochemical activity and molecular modelling. The Class I enzymes possess a single disulphide bridge and contain a variable loop [8], whereas members of the Class II enzymes contain an additional intra-chain disulphide bridge that links a flexible loop with a catalytic loop [8]. Both SMases D classes exhibit differences in their toxic potential: Class II enzymes are less toxic than Class I enzymes [9].

In addition to the *Loxosceles* spiders, other members of the family Sicariidae, such as the genus *Sicarius*, also contain SMases D [3, 10]. Similarly, SMase D toxins were also detected in the genera *Ixodes* and *Rhipicephalus*, in the family Ixodidae [11], and as exotoxins produced by certain pathogenic bacteria. For instance, these toxins were produced by *Corynebacterium ulcerans* and *Arcanobacterium haemolyticum,* which are pathogens that cause pharyngitis and other human infections, and by *Corynebacterium pseudotuberculosis*, which causes lymphadenitis in animals but is also pathogenic for humans [8, 12]. The bacterial and spider venom SMases D possess similar molecular masses (31–35 kDa). However, they show low sequence identity and are considered to have originated from a common ancestor, the glycerophosphodiester phosphodiesterases (GDPD; E.C. 3.1.4.46) [8].

This study addresses both the phylogenetic relations between the structural classes of SMases D and the possibility that the evolution of this harmful protein has been driven by natural selection towards its toxicity. Because the structural classes of *Loxosceles* SMases D were proposed based on the presence on differences of cysteine residues that form disulphide bridges, we hypothesized that this feature may cause some constraints in the evolution of these molecule, as disulphide bridges are expected to interfere in the flexibility/rigidity and, therefore also in the catalytic properties of proteins. To test this hypothesis, we performed a comprehensive phylogenetic analysis of the available SMase D nucleotide sequences in public databanks to test the monophily of structural classes of this enzyme. We also tested amino acids sites and phylogenetic branches to highlight the past natural selection and the evolution within this protein family. *In silico* molecular modelling was applied on the available SMase D crystallographic structure, and the sites associated with natural selection were mapped, to reveal some clues on the structural/functional aspects of these evolutionary changes.

## Methods

### Sequence Analysis

The nucleotide sequences of SMase D enzymes from a total of 29 species (Additional file 1: Table S1) were analysed: a) *Loxosceles* (20 species: *L. hirsuta, L. intermedia, L. deserta, L. arizonica, L. laeta, L. spadicea, L. sabina, L. spinulosa, L. apachea, L. variegata, L. rufescens, L. amazonica, L. reclusa, L. boneti, L.* sp*. 4 GJB-2008, L. aff. Spinulosa GJB-2008, L. gaucho, L. adelaida*, *L. similis*, and, *L. cf spinulosa GJB-2008*); b) *Sicarius* (five species: *S. damarensis* GJB-2008, *S. peruensis, S. patagonicus, S. terrosus* and *S. albospinosus*)*;* c) Bacteria (two species: *Corynebacterium pseudotuberculosis* and *Arcanobacterium haemolyticum*); d) Ticks (two species: *Ixodes scapularis* and *Rhipicephalus pulchellus*). All of the sequences are represented by their GI numbers in Additional file 1: Table S1. The SMase D sequences from spiders (*Loxosceles* and *Sicarius* genera), bacteria (*Corynebacterium pseudotuberculosis* and *Arcanobacterium haemolyticum*) and ticks (*Ixodes scapularis* and *Rhipicephalus pulchellus*) were downloaded from GenBank at the NCBI’s databases (http://www.ncbi.nlm.nih.gov). “Sphingomyelinase D” and “Phospholipase D” were input independently with each species by keyword at the Nucleotide Advanced Search Builder (NCBI). Exclusive Sphingomyelinase D sequences and exclusive Phospholipase D sequences were obtained. Duplicated sequences simultaneously annotated as Sphingomyelinase D and Phospholipase D were computed only once. Moreover, 184 nucleotide sequences of *Loxosceles*’ SMase D were downloaded from GenBank. In these sequences, approximately 1.5 % (3/184) was annotated only as Sphingomyelinase D and 11 % (20/184) was annotated only as Phospholipase D. For 87.5 % (161/184), both annotations were attributed. Expressed sequence tags (ESTs) with high similarities (e-value cut-off = 1e-105) to SMase D were chosen from a previous study from our laboratory about a transcriptome analysis of *L. laeta* spider venom gland [13]. Nucleotides sequences were submitted to the Open Reading Frame (ORF) Finder (http://www.ncbi.nlm.nih.gov/projects/gorf/) to only select flanked sequences by start and stop codons. After excluding the incomplete sequences, 179 (179/184; 97.3 %) nucleotide sequences remained together with fifteen ESTs of high similarity to SMase D. Hence, a total of 194 SMase D unique nucleotide sequences from *Loxosceles* genus were found. For the *Sicarius* genus, all of the 47 downloaded nucleotides sequences were annotated as Sphingomyelinase D and Phospholipase D. After excluding an identical sequence obtained from the ORF finder analysis, 46 (46/47; 97.9 %) sequences were considered. In bacteria, we found three sequences from *C. pseudotuberculosis* and two sequences from *A. haemolyticum*; all of them were annotated as Phospholipase D. In ticks, we found three sequences from *I. scapularis* annotated as Sphingomyelinase D and Phospholipase D and one from *R. pulchellus* annotated as Sphingomyelinase D.

The SMase D’s nucleotide sequences were aligned by MUSCLE with default parameters implemented within MEGA version 6 [14]. The structural classification of SMase D [8] was adopted to classify the SMase D sequences. The enzymes were classified according to the number and position of the cysteine residues that form the disulphide bridges into Class I (cysteine residues in positions analogous to C^51^ and C^57^ of PDB entry 1XX1) [7, 8] or Class II (cysteine residues in positions analogous to C^51^, C^53^, C^57^ and C^201^ of PDB entry 1XX1) [7, 8]. The nucleotide sequences were aligned by codons (MUSCLE) and translated into proteins (MEGA) to classify the isoforms. Incomplete sequences were excluded from further analyses.

### Phylogenetic analyses

The evolutionary relationships among SMase D isoforms from six genera (29 species), *Loxosceles* (twenty species), *Sicarius* (five species), *Arcanobacterium haemolyticum*, *Corynebacterium pseudotuberculosis*, *Ixodes scapularis* and *Rhipicephalus pulchellus* were inferred by nucleotide sequences (Additional file 1: Table S1). Maximum likelihood (ML) and Bayesian inference were used to infer the phylogenetic relationships of aligned non-redundant sequences as implemented at Garli version 2.0 [15] and MrBayes version 3.2.5. [16] The rate variation among sites was modelled with a gamma distribution (shape parameter = 5). The Bayesian inference was performed using nucleotide and codon models. For both ML and Bayesian analyses, the Bayesian Information Criterion (BIC) was applied to evaluate the best model of substitution of nucleotides for tree reconstructions (Find Best DNA Model - MEGA6) [14].

### Estimation of positively selected branches and sites

A subset of aligned SMase D sequences containing all nucleotide sequences from the genus *Loxosceles* was tested for positive Darwinian selection. A tree was obtained with the ML method (GTR + G + I model) from 190 SMase D nucleotides from the genus *Loxosceles* and was manually rooted to be inputted into this analysis using the codeml program from the PAML package version 4.6 [17]. To investigate whether there were different evolutionary rates in these sequences, we performed three approaches using the ML method based on a version of the codon-substitution model [18] implemented in the codeml program (PAML 4.6) [17]: i) branch models; ii) site models and iii) branch-site models. The clean data parameter was adjusted to 1, and the rooted tree was estimated by ML with the best model (Find Best DNA Model) [14]. The selective pressure can be measured by the nonsynonymous (d_N_)/synonymous (d_S_) rate ratio (ω = d_N_/d_S_). Purifying (or negative) selection, neutral evolution and diversifying selection (or positive) are indicated by ω < 1, ω = 1 and ω > 1, respectively. In the branch model analysis, the ω ratio varies among branches in the phylogeny and is designated to detect positive selection acting on particular lineages. The following indicated models [19] were used in our analysis: i) one-ratio model (assumes the same ω ratio for all branches); ii) free-ratio model (assumes an independent ω ratio for each branch); iii) two-ratio model (assumes that the indicated branches have a ω ratio that is different from the background ratio, ω_0_); iv) three-ratio model (assumes that the ratios for different branches, e.g., ω_1_; ω_2_, are different between them and different from the background ratio, ω_0_). In addition, we directly examined the possibility of the occurrence of positive selection with the two- or three-ratio models by assuming values of ω = 1 in the branches of interest.

In the site model analysis, the ω ratio varies among sites (among codons) and is designated to test for the presence of positively selected sites and to identify such sites along the gene [20]. We tested the following recommended site models [21, 22]: i) M0 (one-ratio model, assumes one ω ratio for all coding sites); ii) M1a (nearly neutral model; assumes two ω ratio values 0 < ω_0_ < 1 estimated from the data and ω_1_ = 1, neutral sites, is fixed); iii) M2a (positive selection; allow an additional class to M1a, ω_2_ > 1); iv) M3 (discrete model; assumes a general discrete model, with frequencies and ω ratios for K site classes estimated as free parameters); v) M7 (beta model; consists of a null model that does not allow for positively selected sites; the ω ratio varies within the interval (0,1) and according to the beta distribution with parameters *p* and *q*); vi) M8 (beta & ω; adds an additional class of sites under positive selection to M7 and with ω > 1). The branch-site models allow ω to vary both among sites at the enzyme sequence and across branches on the tree, which aims to detect positive selection affecting a few sites along a branch under test for positive selection (*foreground* branch). Test 2, the branch-site test of positive selection, compares model A [23, 24] with model A with ω_2_ = 1 fixed (null model).

Different models (branch models, site models or branch-site models) can be compared with the likelihood ratio tests (LRTs) to investigate interesting hypotheses. For branch models, we compared the one-ratio and free-ratio models to test whether the ω ratios are different among lineages. We also compared the one- and two-ratio models to investigate whether the lineages of interest have a different ω ratio from other lineages. To directly test the possibility of positive selection on specific lineages, we compared the two-ratio model with and without the constraint of ω ≤ 1 to verify whether the branches of interest have a ω > 1. For the site models, we compared three model pairs by LRT – i) M1a and M2a; ii) M0 and M3, and iii) M7 and M8 – considering that only M2a, M3 and M8 allow for the presence of positive selected sites (ω > 1). For the branch-site models, we use Test 2, the branch-site test of positive selection, to compare Model A [23, 24] with model A with ω_2_ = 1 fixed (null model). LRTs compare two nested models: a null model that assumes no selection (ω ≤ 1) versus an alternative model that allows for positive selection (ω > 1). Twice the log likelihood difference between the two nested models (2Δl) is compared with a χ^2^ distribution with a number of degrees of freedom equal to the difference in the number of free parameters to obtain a p-value for this LRT. The p-values were calculated using the “Chi-square distribution calculator” tool from http://keisan.casio.com/exec/system/1180573196. The BEB approach (CODEM/PAML4.6) [17] was used to identify sites under positive selection.

In addition to the analyses done with the help of the PAML program to identify sites under positive selection, this was also done under the Bayesian approach by using the MrBayes software. All 249 nucleotide sequences were analysed. This was accomplished with the use of a codon based model, NJ98 [25] as implemented in the MrBayes software, to verify the proportions of sites under negative selection (ω < 1), under neutral evolution (ω = 1), and positive selection (ω > 1). This was done by interactively increasing the number of MCMC (Markov chain Monte Carlo) generations until the split standard deviations between simultaneous runs dropped to values below 0.05.

### Statistical analysis

Sites under positive selection found in Classes I and II were subjected to further statistical analysis to investigate whether there were differences between the structural classes of SMase D based on the quantity of disulphide bridges. Each of the possible twenty amino acids found in each SMase D class was assessed with two approaches: i) separately for each of the seven positively selected sites and ii) by the sum of the seven positively selected sites. The data were subjected to the D’Agostino’s test to investigate whether the pairs of values followed a Gaussian distribution. According to this analysis, the two paired groups for each site were compared with both t and Wilcoxon tests. All of the amino acids found in each positively selected site were placed into four categories of both SMase D classes: nonpolar, neutral, acidic, and basic. To investigate whether the difference between (nonpolar plus basic) and (neutral plus acidic) in the two SMase D classes was statistically significant, the data were analysed with a Fisher’s exact test. All of the statistical tests were performed with GraphPad Prism version 5.01 for Windows (GraphPad Software, San Diego California USA, www.graphpad.com).

### *In silico* molecular modelling

The three-dimensional structure of Class II SMases D was modelled using the HHPred server [26], which employs a restraint-based modelling routine from the Modeller program [27]. The atomic coordinates of SMase I, a Class I SMase D from *L. laeta* [7], were used as a template for the *in silico* modelling. The stereochemistry was improved using spatial restraints and CHARMM energy terms, followed by a conjugate gradient simulation, as implemented in the Modeller software [28]. The overall and local quality analyses of the final model were assessed with VERIFY3D [29], PROSA [30] and VADAR [31]. The atomic models were analysed and compared using the PyMol program (http://www.pymol.org).

## Results

### Classification of SMase D sequences

**Fig. 1:**
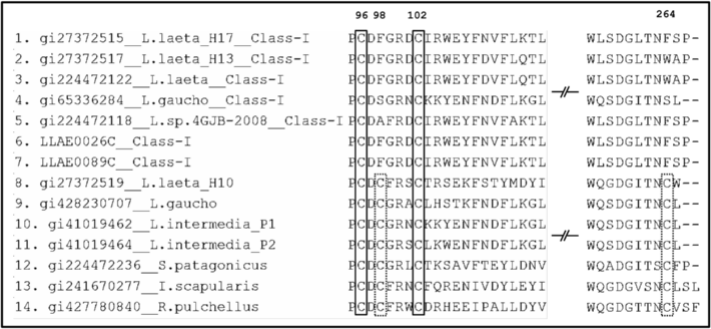
Partial multiple alignment of amino acid sequences of SMase D. In this analysis, sites Cys^96^ and Cys^102^ correspond to Cys^51^ and Cys^57^, respectively, and form a disulphide bridge in Class I and Class II isoforms. The sites Cys^98^ and Cys^264^ (corresponding to Cys^53^ and Cys^201^) form an additional bridge in SMase D Class II members

Nucleotide SMase D sequences from 29 species, including *Loxosceles* spp. (twenty species), *Sicarius* spp. (five species), ticks (two species) and bacteria (two species), were obtained from databanks (Additional file 1: Table S1). Subsequently, 249 nucleotide sequences were translated and classified into Class I or Class II based on the structural classification (Additional file 1: Table S1). It was the first structural classification of all available sequences from SMase D. Figure 1 shows the number and position of cysteine residues that form the disulphide bridges in certain representative sequences. In Class I enzymes, there is only one disulphide bridge, between Cys^96^ and Cys^102^ (corresponding to Cys^51^ and Cys^57^, according to the PDB entry) [7]. In Class II enzymes, there is an additional ligation between Cys^98^ and Cys^264^ (corresponding to Cys^53^ and Cys^201^, according to the PDB entry) [7]. A structural classification analysis indicated that nineteen nucleotide sequences (19/249; 7.6 %) belong to the Class I enzymes, and 225 nucleotide sequences (225/249; 90.4 %) belong to the Class II enzymes. Approximately 2 % of the sequences (5/249 nucleotides) correspond to bacterial Phospholipase D, which does not present cysteine residues at homologous sites compared with other sequences; thus, these sequences were not classified. Interestingly, Class I enzymes (approximately 8 %) are present only in *L. laeta*, *L. gaucho* and *L. sp. 4 GJB-2008*, whereas Class II isoforms (approximately 90 %) are more abundant and only absent in *Loxosceles* sp*. 4 GJB-2008*. For this latter species, only two Class I isoforms were found to date (Additional file 1: Table S1).

### Phylogeny of structural classes

**Fig. 2:**
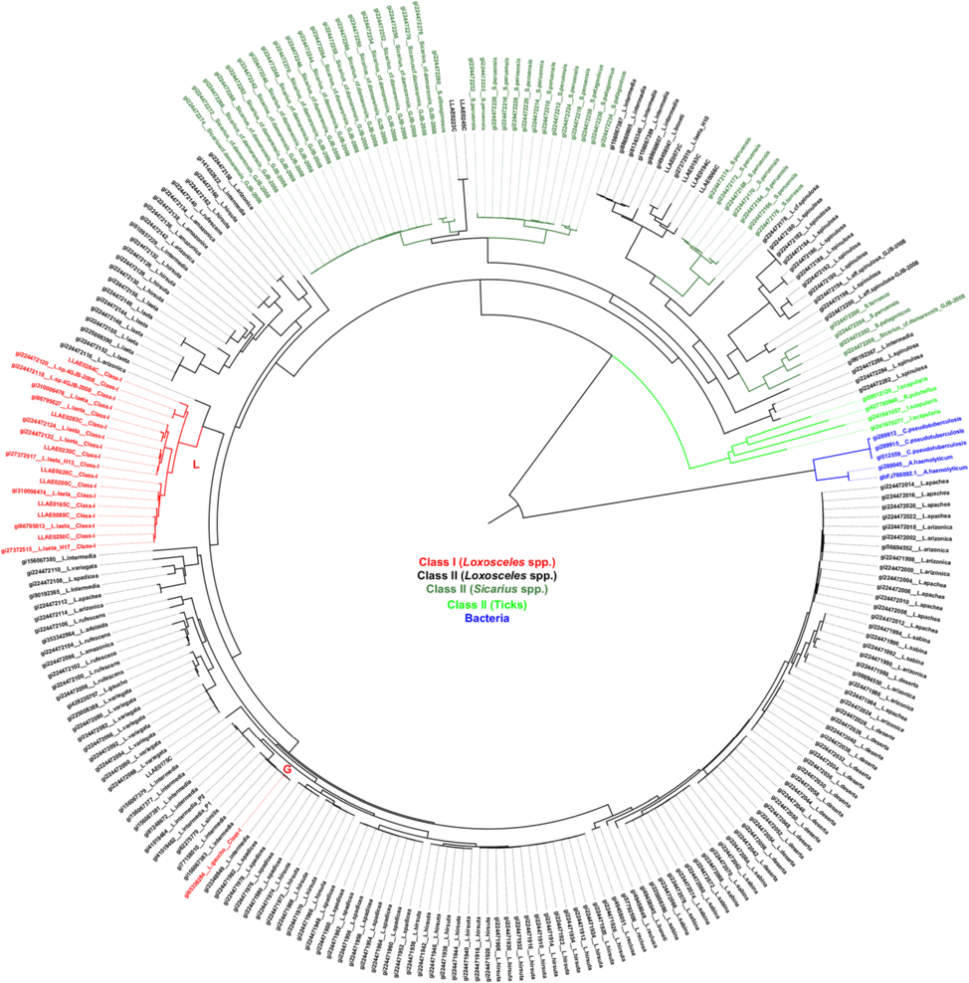
Phylogenetic analysis of SMase D sequences by ML method. Phylogenetic tree based on the ML method (lnL = −46,243.0294). A GTR model was chosen (BIC score = 72,153; lnL = −33,053.48462). Gamma distribution (G parameter = 2.5232). The rate variation model allowed certain sites to be evolutionarily invariable (*I*). *Loxosceles* SMase D Class I isoforms are indicated in clades L and G (red)

**Fig. 3:**
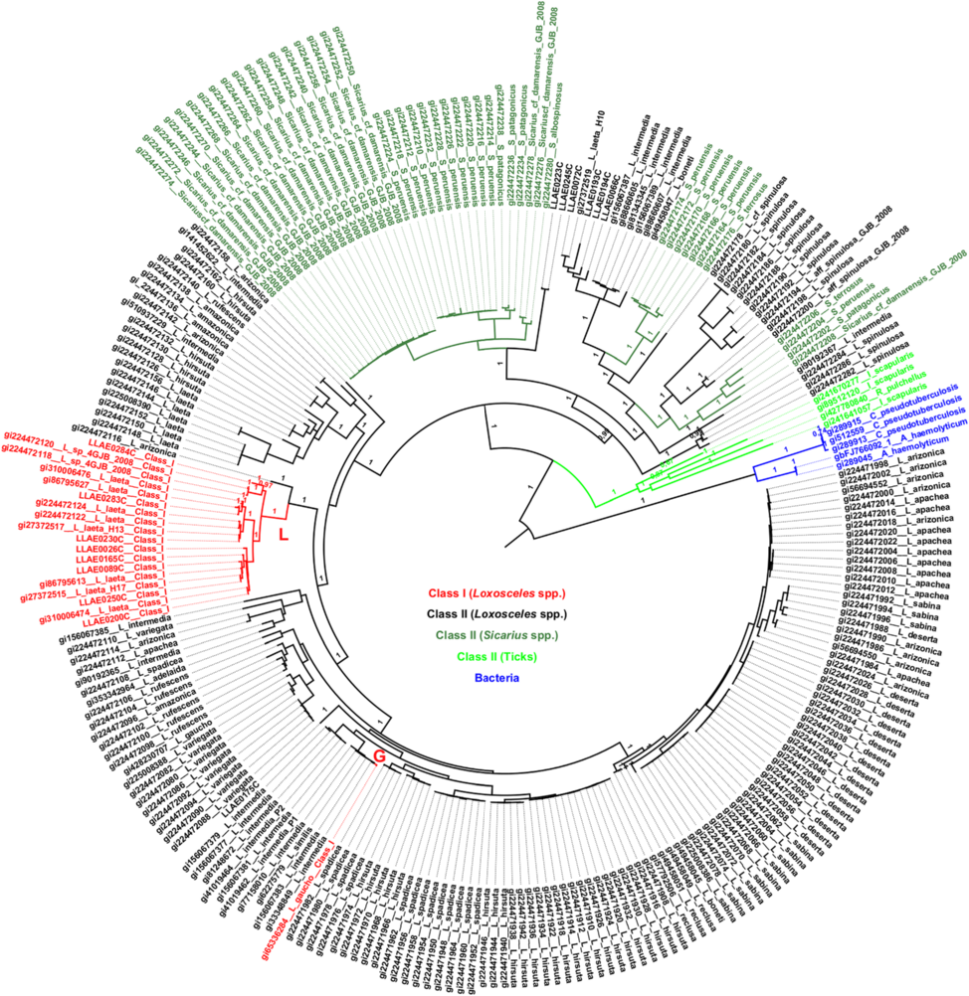
Phylogenetic analysis of SMase D sequences by nucleotide model. Bayesian inference tree based on the nucleotide model. A GTR model was chosen (BIC score = 72,153; lnL = −33,053.48462). Gamma distribution (G parameter = 2.5232). The rate variation model allowed certain sites to be evolutionarily invariable (*I*). Posterior probabilities are indicated. *Loxosceles* SMase D Class I isoforms are indicated in clades L and G (red)

**Fig. 4:**
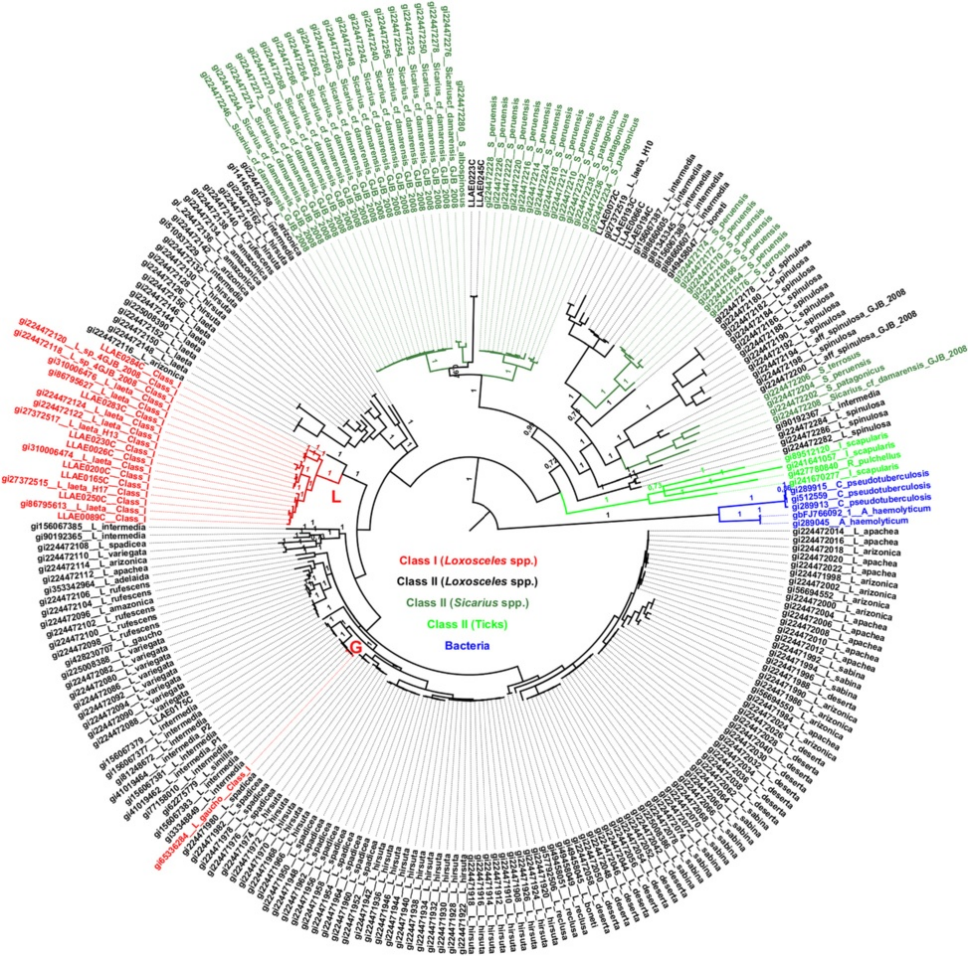
Phylogenetic analysis of SMase D sequences by codon model. Bayesian inference tree based on the codon model. A GTR model was chosen (BIC score = 72,153; lnL = −33,053.48462). Gamma distribution (G parameter = 2.5232). The rate variation model allowed certain sites to be evolutionarily invariable (*I*). Posterior probabilities are indicated. *Loxosceles* SMase D Class I isoforms are indicated in clades L and G (red)

The General Time Reversible (GTR) model with the lowest Bayesian Information Criterion (BIC) score (BIC = 72,153; lnL = −33,053.48462) was chosen to describe the best substitution pattern by the ML method and Bayesian inference. A discrete Gamma distribution was used to model the evolutionary rate differences among sites (5 categories; G parameter = 2.5232). The tree with the highest log likelihood (−46,243.0294) for ML method is indicated in Fig. 2. The Bayesian inference tree for nucleotide model is indicated in Fig. 3 and for codon model in Fig. 4. In all approaches, the Class I isoforms were grouped in two clades, referred here as clades L and G (Figs. 2, 3, and 4). Clade L includes eighteen isoforms from two species, *L. laeta* and *L. sp. 4 GJB-2008*. Clade G includes only one *L. gaucho* sequence.

The phylogenies inferred by the ML and Bayesian methods were almost identical. The few (less than 5 %) differences were located in very short branches. The codon based analysis that was performed with MrBayes to assess sites under positive selection produced a consensus tree that was also very similar to those obtained so far, except to minor differences in the topologies also involving very short branches (Fig. 4).

### Detection of positive selection on SMase D

Because Class I SMase D enzymes were grouped into two clades, we investigated the presence of positive Darwinian selection in the evolution of these enzymes. Three approaches were employed: branch models, site models and branch-site models with the use of the PAML package. To detect positive selection acting on Class I SMase D isoforms present in clades L and G (Figs. 2, 3 and 4), eleven branch models (A-K; Table 1) were tested. The models attribute different restriction on three d_N_/d_S_ ratio parameters: ω_L_ for clade L, ω_G_ for clade G, and ω_0_ for background branches. The models may assume one d_N_/d_S_ ratio (A and F; Table 1), two ratios (B-D and F-H), three ratios (E, I, and J), and an independent d_N_/d_S_ ratio for each branch (free-ratio model; K). The log-likelihood values of different models are given in Table 1. The estimate of the d_N_/d_S_ ratio under the one-ratio model (ω_0_ = ω_L_ = ω_G_) was 0.254, which indicates that synonymous substitution occurred more often than nonsynonymous substitutions and that SMase D has spent time under negative purifying selection during the *Loxosceles* evolution. Estimates of ω_L_ for clade L and ω_G_ for clade G were approximately 0.47 (B, E and J; Table 1) and 1.0, respectively, when they were free to vary. When the same ratio is assumed for clades L and G (ω_L_ = ω_G_; model D), the estimate was 0.47, which is an average of the two branches. When ω_L_ and ω_G_ are not restricted to be equal to ω_0_ (models D, E, I and J), the estimated background ratio (ω_0_) was 0.24.

**Table 1:** Log Likelihood (l) values and parameter estimates under different models (branch models) in PAML

Model	l	ω_0_	ω_L_	ω_G_
A. One Ratio: ω_0_ = ω_L_ = ω_G_	−28843.35	0.254	ω_0_	ω_0_
B. Two Ratios: ω_0_ = ω_G_; ω_L_	−28825.10	0.241	0.466	ω_0_
C. Two Ratios: ω_0_ = ω_L_; ω_G_	−28842.52	0.254	ω_0_	0.996
D. Two Ratios: ω_0_; ω_L_ = ω_G_	−28824.44	0.241	0.469	ω_L_
E. Three Ratios: ω_0_; ω_L_; ω_G_	−28824.20	0.241	0.466	0.997
F. Two Ratios: ω_0_ = ω_G_; ω_L_ = 1	−28848.59	0.241	1.000	ω_0_
G. Two Ratios: ω_0_ = ω_L_; ω_G_ = 1	−28842.71	0.254	ω_0_	1.000
H. Two Ratios: ω_0_; ω_L_ = ω_G_ = 1	−28847.69	0.241	1.000	1.000
I. Three Ratios: ω_0_; ω_G_; ω_L_ = 1	−28848.56	0.241	1.000	1.000
J. Three Ratios: ω_0_; ω_G_ = 1; ω_L_	−28824.20	0.241	0.466	1.000
K. Free Ratio	−28505.20	---	---	---

Likelihood ratio tests (LRTs) were applied to compare different models to test several hypotheses (Table 2). First, we compared a one-ratio model and a free-ratio model to test whether the d_N_/d_S_ ratios are different among lineages (Test F; Table 2). The likelihood scores (2Δl = 675.84) and the correspondent p-value (5.99x10^−56^) permitted the rejection of the null hypothesis (ω_0_ = ω_L_ = ω_G_), which indicates that the d_N_/d_S_ ratios were different among lineages. To examine whether the d_N_/d_S_ ratio for clade L (ω_L_) and clade G (ω_G_) was greater than the background ratio (ω_0_), we tested hypotheses A-E. Similarly, we tested hypotheses A’-E’ to determine whether these ratios were greater than one (Table 2). In hypotheses A and A’, clades L and G were assumed to have the same d_N_/d_S_ ratio (ω_L_ = ω_G_). This ratio was significantly greater than the background ratio ω_0_ (*P* = 7.8x10^−10^; Table 2; A) and significantly greater than one (*P* = 9.18x10^12^; Table 2, A’). Tests B-E and B’-E’ revealed which of the clades, L or G, were responsible of significant results. ω_L_ and ω_G_ were allowed to differ, and in each case, only one was compared with the background ratio ω_0_; the other could vary freely or could be restricted to be equal to ω_0_. The results indicate that ω_L_ was significantly greater than the background ratio ω_0_ (*P* = 1.53x10^−09^ for test B and *P* = 7.29x10^−09^ for test C; Table 2) and also significantly greater than one (*P* = 7.15x10^−12^ for test B’ and *P* = 2.96x10^−12^ for test C’; Table 2), although ω_L_ ratio was 0.47. However, ω_G_ was neither significantly greater than one nor greater than the background ratio ω_0_ (*P* ranges from 18 % to 99 %; Table 2, D, D’, E, E’). The LRTs from the branch models analysis indicate that clade L, but not clade G (Figs. 2, 3 and 4), is under weaker negative selection, as ω_L_ was significantly different between clade L and the background level (Table 2).

**Table 2:** Likelihood scores (2Δl) for testing hypotheses for *branch models* in PAML

Null Hypothesis	Assumption made	Models compared	2Δl	*P*
A. (ω_L_ = ω_G_) = ω_0_	ω_L_ = ω_G_	A X D	37.81	7.81 x 10^−10^
B. ω_L_ = ω_0_	ω_G_ = ω_0_	A X B	36.50	1.53 x 10^−09^
C. ω_L_ = ω_0_	ω_G_ free	C X E	36.64	7.29 x 10^−09^
D. ω_G_ = ω_0_	ω_L_ = ω_0_	A X C	1.65	1.99 x 10^−01^
E. ω_G_ = ω_0_	ω_L_ free	B X E	1.78	1.81 x 10^−01^
F. ω constant	No Free Ratio	A X K	675.84	5.99 x 10^−56^
A’. (ω_L_ = ω_G_) ≤ 1	ω_L_ = ω_G_	D X H	46.50	9.18 x 10^−12^
B’. ω_L_ ≤ 1	ω_G_ = ω_0_	B X F	46.99	7.15 x 10^−12^
C’. ω_L_ ≤ 1	ω_G_ free	E X I	48.72	2.96 x 10^−12^
D’. ω_G_ ≤ 1	ω_L_ = ω_0_	C X G	0.37	5.50 x 10^−01^
E'. ω_G_ ≤ 1	ω_L_ free	E X J	2e-05	9.96 x 10^−01^

A site model analysis allows the ω ratio to vary among sites (among codons or amino acids). The following recommended models [21, 22] were applied: M0 (one ratio), M1a (nearly neutral), M2a (positive selection), M3 (discrete), M7 (beta) and M8 (beta & ω). The LRT of the M0-M3 pair was used as a test of variable ω among sites rather than a test of positive selection. The LRT of positive selection comprises two pairs of models: i) M1a and M2a and ii) M7 and M8. Table 3 shows the results with the statistics of likelihood ratio (2Δl) for the hypotheses tested. The M0-M3 test indicates that there were variable ω among sites (2Δl = 1666.50; df = 4; *P* = 1.107x10^−359^). The M1a-M2a (2Δl = 14.56; df = 2; *P* = 6.87x10^−4^) and M7-M8 (2Δl = 35.54; df = 2; *P* = 1.92x10^−8^) pairs suggest that there were certain sites under selective pressure in the SMase D isoforms from *Loxosceles*. Four positively selected sites were detected by the M1a-M2a pair (75.4 % ≤ P ≤ 91.1 %), and seven positively selected sites were detected by the M7-M8 pair (52.3 % ≤ P ≤ 99.9 %).

**Table 3:** Parameter estimates under different site models and statistics of the likelihood ratio (2Δl) hypothesis tests in PAML

Model	Estimates of parameters	- l	LRT (pairs)	df	2Δl	*P*	Positively selected sites BEB (%)	
M0: one ratio	ω = 0.25443	28843.35	M0/M3	4	1666.50	1.11 x 10^−359^	N.A.	
M3: discrete	ω_0_ = 0.03317; ω_1_ = 0.22423; ω_2_ = 0.62748; p_0_ = 0.26344; p_1_ = 0.47946; p_2_ = 0.2571	28010.09					None	
M1a: neutral	ω_0_ = 0.18188; ω_1_ = 1.00000; p_0_ = 0.73346; p_1_ = 0.26654	28338.75	M1/M2	2	14.56	6.87 x 10^−04^	NA	
M2a: selection	ω_0_ = 0.184690; ω_1_ = 1.000000; ω_2_ = 1.879270; p_0_ = 0.72964; p_1_ = 0.25444; p_2_ = 0.01592	28331.46					58I (99.1 %); 204L (94.0 %); 208A (75.4 %); 236S (80.0 %)	
M7: beta	p = 0.70821; q = 1.65979	27977.46	M7/M8	2	35.54	1.92 x 10^−08^	NA	
M8: beta & w	p_0_ = 0.96464; p = 0.85258; q = 2.56188; p_1_ = 0.03536; ω = 1.262270	27959.69					18K (67.8 %); 38K (53.0 %), 58I (99.9 %); 151K (52.3 %)	204L (98.2 %); 208A (92.3 %); 236S (95.5 %)
Model A (null)	p_0_ = 0.52400; p_1_ = 0.18218; p_2a_ = 0.21802; p_2b_ = 0.07580; ω_0_ = 0.16817; ω_1_ = 1.00000; background: ω_2a_ = 0.16817; ω_2b_ = 1.00000; foreground: ω_2a_ = 1.00000; ω_2b_ = 1.00000	28279.91	Null/ Alt.	1	13.89	1.94 x 10^−04^	21P (95.7 %); 22T (100.0 %); 40S (99.0 %); 53F (98.7 %); 84G (97.2 %); 144G (99.8 %); 179T (98.5 %); 201F (98.2 %)	205G (99.8 %); 214I (95.3 %); 231S (100.0 %); 239R (95.9 %); 240K (97.2 %); 243E (100.0 %); 253Y (99.6 %); 265G (99.8 %)
Model A (alternative)	p_0_ = 0.58498; p_1_ = 0.20363; p_2a_ = 0.15681; p_2b_ = 0.05458; ω_0_ = 0.17009; ω_1_ = 1.00000; background: ω_2a_ = 0.17009; ω_2b_ = 1.00000; foreground: ω_2a_ = 1.97553; ω_2b_ = 1.97553	28272.97					NA	

l = lnL = log likelihood; LRT: likelihood ratio test; df: degrees of freedom; 2Δl: twice the log-likelihood difference of the models compared. *P*: p-value; BEB: Bayes Empirical Bayes (BEB) probabilities

**Fig. 5:**
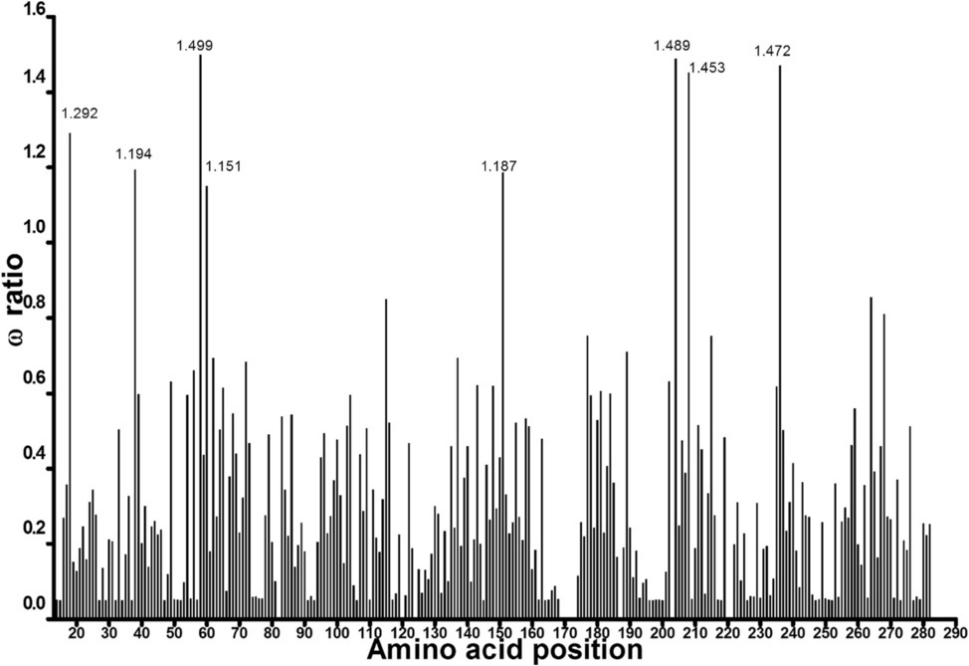
Estimates of ω ratios for sites in the SMase D enzymes. Positively selected sites (ω > 1) for both SMase D classes are indicated

Figure 5 shows the relationship between the amino acid position and their respective dN/dS (ω) ratio values obtained after comparing the M7-M8 pair by the LRT. Only sites 58I, 204L and 236S showed posterior probabilities greater than 95 % (Table 3). To identify the exclusive sites under positive selection in Class I SMase D, we specified clade L as the *foreground* branch and all other branches of the tree as the *background* branches. Test 2 for branch-site models revealed that there were sixteen positively selected sites on clade L (2Δl = 13.89; df = 1; *P* = 1.94x10^−04^; Table 3). Seven sites were detected with P ≤ 1 %, of which three had *P* = 0 and nine sites had 1 % < P ≤ 5 % (Table 3). The posterior probabilities values were obtained by the Bayes Empirical Bayes (BEB) method. The Bayesian approach as implemented in MrBayes to detect the proportion of sites under different types of selection, after 200,000 MCMC generations, resulted in the following proportions (values between parentheses indicate the results of another, independent run): 76.0 % (76.9 %) negatively selected sites, 22.7 % (21.7 %) neutrally evolving sites, and 1.3 % (1.3 %) positively selected sites. Among the top eight sites that presented the highest ω, seven sites were also found to be under positive selection with the ML approach (Table 4).

**Table 4:** Top most positively selected sites according to the Bayesian approach with MrBayes

Site	Amino acid	ω (mean)	P (ω > 1.0)
18 *	K	1.00094	0.0419
38 *	K	1.00106	0.0426
58 *	I	1.00190	0.0466
151 *	K	1.00090	0.0416
204*	L	1.00400	0.0528
208 *	A	1.00320	0.0520
236 *	S	1.00170	0.0450
268	D	1.00125	0.0438

* sites that were also found to be positively selected with the ML approach

### Amino acids frequencies in sites under adaptive evolution

**Fig. 6:**
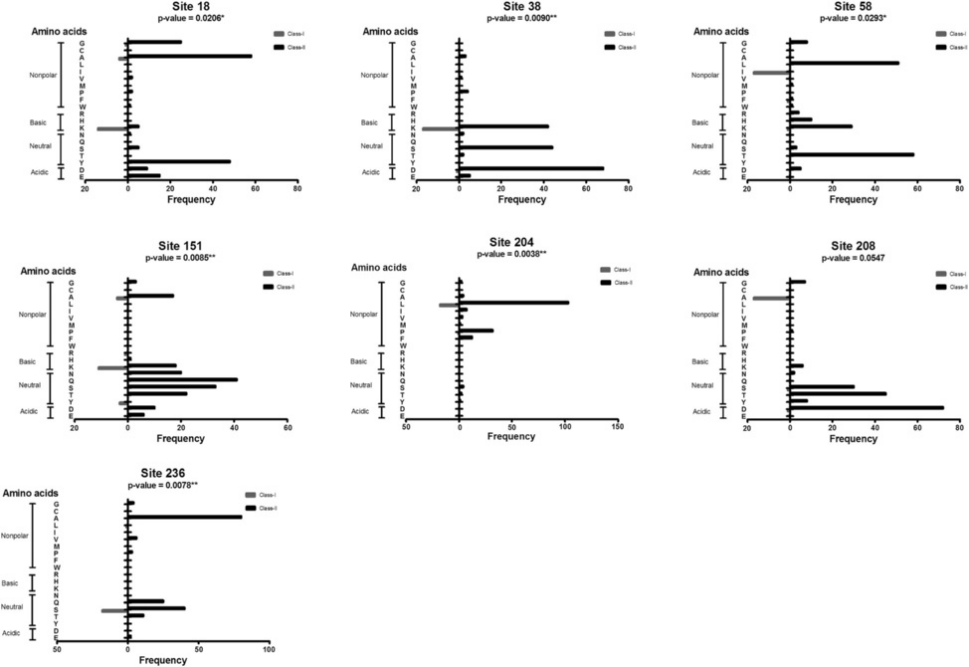
Statistical analysis of SMase D classes for each site under positive selection. The absolute frequency of amino acids found in each positively selected site is indicated for Classes I and II. Nonpolar, basic polar, neutral polar and acidic polar amino acids are indicated. The p-values obtained by the Wilcoxon test are shown

**Fig. 7:**
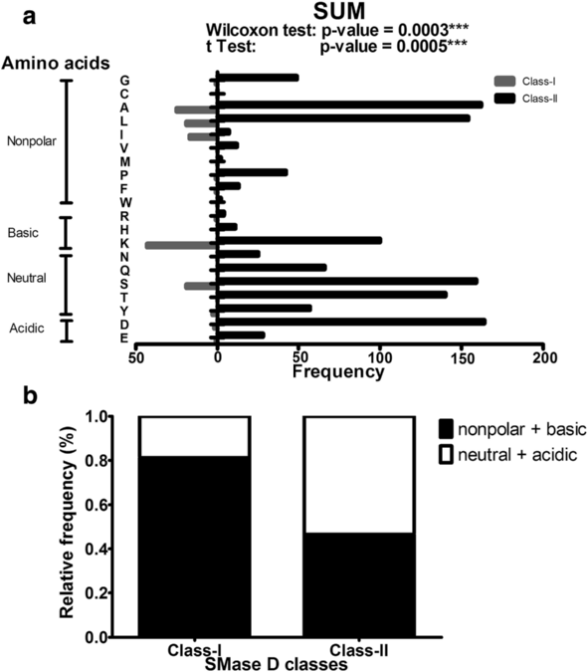
**a** Statistical analysis of SMase D classes for sum of sites under positive selection. The absolute frequency of summed amino acids is indicated for Classes I and II. Nonpolar, basic polar, neutral polar and acidic polar amino acids are indicated. The p-values obtained by the Wilcoxon test and t test are shown. **b** Distribution of nonpolar + basic and neutral and acidic residues in Class I and Class II members

Seven sites under positive selection were detected (Table 3). They belong to both SMases D classes. We investigated whether there was any difference in positive selected sites between Classes I and II regarding the frequencies and types of amino acids. A statistical analysis of each site was performed individually (Fig. 6) or summed (Fig. 7a), which indicate that the distribution of amino acids under selection, except for site 208, is different between classes (P < 0.05). The categories of amino acids (nonpolar and polar as well as basic, neutral and acidic) were analysed (Fig. 7b). The statistical analysis shows that nonpolar and basic residues are more abundant in sites under positive selection of Class I members and neutral and acidic are more abundant in sites under positive selection of Class II members (Table 5).

**Table 5:** Statistical analysis of amino acids classes found in SMase D positively selected sites. Fisher's exact test

	nonpolar + basic	neutral + acidic	Total
Class-I	108	25	133
Class-II	558	639	1197
Total	666	664	1330

*P* value < 0.0001

### Sites on the SMase D three-dimensional structure

**Fig. 8:**
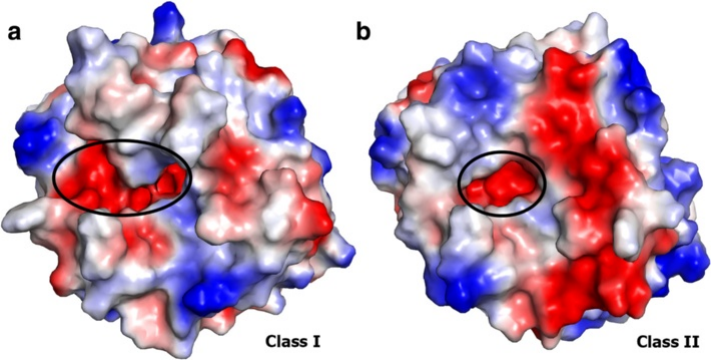
Structural interpretation of the positively selected sites. **a** Cartoon representation of the structural comparison between Class I and II SMases D. The fully conserved catalytic histidines (H12 and H47) and the three acidic residues (E32, D34 and D91) involved in the metal ion coordination are shown as sticks, with carbon atoms in green. The seven positively selected sites are shown as sticks and balls, with carbon atoms in yellow. The residues depicted in the positive sites correspond to those of SMase I from *L. laeta*, and the sequence numbering is also based on this molecule according to PDB entry 1XX1 (Murakami et al., 2005). The cartoon representation is coloured according to the secondary structure elements and the flexible loop F related to the second S-S bond found uniquely in Class II members (in orange, Class I; in red, Class II). The variable loop E is cyan and blue for Classes I and II, respectively. **b** Schematic representation of a Class I SMase D highlighting all of the positively selected sites for this class using the same colour pattern

**Fig. 9:**
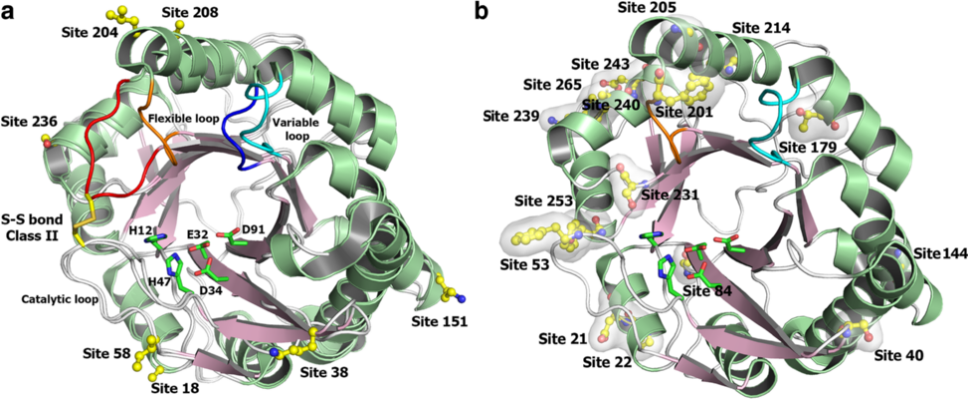
Electrostatic surface charge distribution of Class I and II SMases D highlighting the catalytic interface. The ellipses indicate the active-site pocket. **a** Class I; **b** Class II

A comparative structural analysis of the electrostatic surface charge between Class I and II members supports the predominance of basic residues in Class I, whereas acidic residues are more abundant in Class II enzymes. As shown in Fig. 8, the Class II members have a long negative patch in the catalytic interface. In addition, the active-site pocket of Class II members is partially occluded by a disulphide bridge between the flexible and catalytic loops (Fig. 9a), which restricts the accessibility and size of the substrate [7, 8].

Interestingly, all seven positive sites detected by a site model method were found on the surface of the protomer and contribute to the delineation of the catalytic interface (Fig. 9a). For instance, site 58 is located in the catalytic loop, and site 38 is at the C-terminal end of the β strand, which contains two acidic residues (Glu^32^ and Asp^34^; Fig. 9a). Site 236 is adjacent to the flexible loop, and sites 204 and 208 are located in the α-helix that precedes this loop. Surprisingly, only site 38 was located on the beta strand; other sites were mainly located in the alpha-helix and the loop region (Fig. 9a). For the Class I enzyme, we found 10/16 (62.5 %) sites localized on alpha helixes and 6/16 (37.5 %) located on loop regions (Fig. 9b). Sites under positive selection were not found on beta strands. We found positively selected sites next to the active site, metal-binding sites, in the neighbourhood of important residues of catalytic activity and in the sites 53 and 201of Class I SMase D, which, in Class II, ascribe an additional disulphide bridge. In the GDPD domain (PDB residues: 226–255), we found sites under positive selection that belong exclusively to clade L (231, 239, 240, 243 and 253). Similarly, site 236, which belongs to both classes, is under positive selection.

## Discussion

### How the different structural classes did originate in the evolutionary history of the spider SMases D?

SMases D are the principal molecules responsible for the toxicity presented by spider venoms from genus *Loxosceles*. This enzyme also is present in the spider genus *Sicarius*, in ticks (*Ixodes scapularis* and *Rhipicephalus pulchellus*) and in strains of *Arcanobacterium* and *Corynebacterium*. Based on the sequence alignment, biochemical and structural data, Murakami et al. [8] proposed a structural classification of spider venom SMases D. Class I SMase D is characterized by a single disulphide bridge and an extended hydrophobic loop, while Class II SMase D contains an additional intra-chain disulphide bridge that links the shortened flexible loop with catalytic loop. Class II enzymes can be further subdivided into Class IIa and Class IIb, where only the first is capable of hydrolyzing sphingomyelin. Faced with this, firstly we asked what would be the present distribution of classes in the databank and secondly, what is the evolutionary significance of structural classification of SMase D.

The sample we analyzed was constituted by all available Sphingomyelinase D sequences available in public databases at the moment of the retrieval. The huge majority was classified as Class II. The sequences of SMase D isoforms from *Loxosceles* were obtained by two approaches. In *L. laeta* and *L. intermedia* they were found in transcriptomes of the venom gland[13, 32]. In these species, only *L. laeta* presented Class I and Class II isoforms. The other sequences were obtained by sequencing cloned PCR products with heterologous primers ([10, 33] and Genbank). Except for *L. laeta*, *L. gaucho* and an unidentified species (*L.* sp*. GJB-2008*), all the sequences obtained by this PCR based method were classified as Class II only. There is, in the total of all sequences available for *L. laeta* and *L. gaucho*, an approximate ratio of 50 % for each class (Additional file 1: Table S1), so we would expect to find this class in other species if they are present in similar proportions, what was not the case. Therefore we assumed that, if Class I isoform were present, they would be found. Of course, if further research on sequencing SMase D from other *Loxosceles* species shows that there are other species with Class I isoforms present, the conclusions here drawn should be modified accordingly.

A previous phylogenetic study on the SMase D of spiders, including the genera *Loxosceles* and *Sicarius*, identified two major clades [10]: clade α contains only SMase D sequences of venoms from New World *Loxosceles* and *Loxosceles rufescens*, and clade β includes toxins from New World *Loxosceles*, *Sicarius* and African *Loxosceles*. To address the positioning of structural classes in the phylogeny of these enzymes, nucleotide sequences were subjected to a phylogenetic analysis. The ML and Bayesian phylogenies revealed that Class I SMases D from *L. laeta and L.* sp. *GJB-2008* belong to same clade (clade L), which indicates the recent evolution from a common ancestor.

Clades L and G indicate that Class I SMase D have polyphyletic origins. The use of phylogenies based on molecules that may be subject of selection are considered to be potentially misleading to the hypothesis of species phylogeny, when compared to those based on neutrally evolving molecules. However, if there is convergent evolution, the distorted result on the phylogeny topology would be to infer monophyly instead of polyphyly, if the number of involved residues is relatively high. The presence of Class I SMase D in different clades, however, seems to be the result of convergent evolution of the double disulphide bond in the molecule. Bacteria and ticks were found as monophyletic groups; however, *Loxosceles* and *Sicarius* genera were found to be polyphyletic groups. Other studies on SMase D Bayesian analyses also indicate that *Loxosceles* and *Sicarius* genera are not monophyletic groups [10]. However, duplications and losses of genes prevent SMase D from being the best marker to investigate species relationships. These results also indicate that the single disulphide bridge is a condition that appeared independently in the two clades of the SMase D phylogeny. These results are the first to indicate that the increase in disulphide bond number is a convergent pattern.

### Is there positive selection on SMase D?

The comparison of synonymous (d_S_) and nonsynonymous (d_N_) substitution rates by the ω ratio (ω = d_N_/d_S_) is an indicator of the selective pressure acting on a protein-coding gene [19, 20, 22–24, 34]. Positive selection (diversifying selection) occurs when ω > 1 [19–24, 34]. However, in cases where adaptive evolution occurs in a short period of time affecting a few amino acids, the ω ratio may not be significantly >1. According to different studies [19–24, 34], our first approach to address this problem consisted in the analysis of branch models, site models, and branch-site models. Positive selection was not found on SMase D only in branch models analysis, probably because its power of detection. Also the signal may have been masked by purifying selection. The positively selected sites found by site models and branch-site models were located on the SMase D three-dimensional structure (see below). The sites found in the clade L may indicates a diversification of the protein function in Class I SMase D that could be correlated with the difference in toxic potential between Classes I and II. Our second approach, by Bayesian analysis of all available sequences, allowed proving the sites detected by site models (codeml).

The two analyses performed to verify the sites under positive selection considering the full phylogeny pointed to almost the same sites. This is not surprising because they both are based on the rate synonymous/non synonymous rate of substitutions that occurred during the evolution of the considered enzymes. The Bayesian approach, despite pointing to the sites with rates higher than 1.0, was much more conservative than the ML approach. Thus, based on these approaches, we can state that SMase D is under positive selection.

### What are the roles of the sites?

To investigate the roles of sites under adaptive evolution, they were mapped into the SMase D three-dimensional structure. We also performed a statistical analysis on the differences in frequencies and types of amino acids between Classes I and II found in the site model analysis.

#### Differences between classes

SMase D can have different structural regions that participate in the recognition, binding and hydrolysis of the substrate [5]. Considering that the outer monolayer of most eukaryotic membranes is composed mainly of zwitterionic phospholipids [35], phosphatidylcholine (PC) and SM, electrostatic interactions may occur between acidic residues in sites under diversifying selection (anionic) of Class II SMase D and residual charges of headgroups choline (cationic) from the outer monolayer. A study on ^31^P-NMR on three SMase D, two from *L. arizonica* and one from *S. terrosus*, showed a strong preference for positively charged headgroups (choline and/or ethanolamine) [36]. Importantly, the isoforms from *S. terrosus* have a strong preference for ethanolamine over choline, and this preference might be relevant for the predatory behaviour because ceramide phosphoethanolamine (CPE) is an important sphingolipid present in the main prey of sicariid spiders, insects [36]. Our results correspond with those results because the three enzymes analysed in that study are Class II, according to the structural classification, and in our analysis, these enzymes have acidic or neutral residues in sites under diversifying selection. Therefore, they may potentially set electrostatic interactions to choline or CPE in the outer surface of plasma membranes. It would be interesting to investigate which is the preference of Class I members by ^31^P-NMR as well. In our analysis, electrostatic interactions between basic residues in sites under positive selection and sialic acid residues at the glycocalyx could be favoured, which would indicate a certain adaptive aspect to Class I enzymes. The gangliosides are the most abundant glycolipids in nerve cells and are also present in smaller quantities in other cell types. Spider SMase D acts on the nervous system of the prey and can kill an insect instantly. This characteristic agrees with our hypothesis of the interaction between gangliosides and SMase D. The hydrophobic interactions between nonpolar residues and the hydrophobic core of the bilayer can be facilitated in Class I members. The constant binding of bee venom, melittin, to the PC bilayers is relatively high, which shows the importance of the hydrophobic interactions in other venoms (reviewed by [37]).

#### Sites in the three-dimensional structure

The three-dimensional structure of SMases D from spider venoms consists of a classical (β/α)_8_-barrel with a Mg^2+^ ion coordinated by the acidic residues (Glu^32^, Asp^34^ and Asp^91^), which are relevant for substrate binding, and two catalytic histidine residues, His^12^ and His^47^ [7, 8] (Fig. 9a). The main differences between Classes I and II reside in the catalytic interface (Figs. 8 and 9).

A structural analysis showed a distinct charge distribution across the catalytic interface with a predominance of acidic residues in Class II members compared to Class I enzymes (Fig. 8). Despite the negatively charged active site pocket, which is related to metal ion coordination, Class II members have a long negative patch in the catalytic interface that might interfere with the absorption in membranes and with the function of the molecule. The positively selected sites are in accordance with this observation because most of these sites (18, 38, 58, 151, 204, 208 and 236) are populating the catalytic interface (Fig. 9a) and thus contributing to the surface charge distribution.

In contrast to the disulphide bond that connects the catalytic loop to the flexible loop only found in Class II members, the insertion of the sequence PYLPSL in the variable loop E is a unique feature of Class I SMases D. This insertion results in a hydrophobic prominence on the surface of the catalytic interface, which likely favours the interaction with membranes. This observation together with the high frequency of basic and nonpolar residues at the positive sites in Class I could be related to the association between Class I SMases D and biological membranes. In contrast, the additional S-S bond in Class II SMases D confers rigidity to the catalytic interface and an altered topology of the active site, which is likely related to a functional differentiation. The GDPD domain includes important residues that participate on substrate recognition in plasma membranes [33]. However, further studies are necessary to understand the role of sites under positive selection in the interaction of the enzyme with cell membranes. Thus, the positively selected sites located on the catalytic interface and neighbouring sites are important for the functioning of the enzyme. Moreover, the enzyme has positively selected sites that are conducive to the interaction with the membranes.

The finding of independent evolutionary origin of at least two Class I SMases D leads to some interesting questions that can be further explored. As *L. laeta* and *L. gaucho* are among the most toxic *Loxosceles* species to humans, a throughout comparison of biochemical properties of this Class I enzymes between these two species would certainly bring interesting results.

## Conclusions

This study demonstrated that Class I SMases D from *L. laeta* and *L.* sp*. GJB-2008* belong to the same clade, which indicates a recent evolution from a common ancestor. Similarly, we found a single disulphide bridge, a condition that evolved independently in two clades of *Loxosceles*. The detection of positive selection in clade L, the higher proportion of nonpolar and basic residues in Class I enzymes and the higher proportion of neutral and acidic residues in Class II enzymes under selection indicates the nature of the adaptive evolution that has occurred during the SMase D phylogenetic history. Among the sites under diversifying selection in Class I enzymes, sites 53 and 201 may prevent the formation of an additional disulphide bridge, and this condition brings certain evolutionary advantage to Class I enzymes. The difference between classes and the differences in selection regimens of sites in clade L indicates a diversification of protein functions in Class I SMase D that could be correlated with differences in the toxic potential between Classes I and II. The identification of the functional site(s) would aid in the design and testing of suitable anti-sphingomyelinase compounds in the development of novel therapies to treat loxoscelism.

### Availability of supporting data

The data sets supporting the results of this article are included within the article and its additional file.

## Additional file

Additional file 1: Table S1. DNA sequences from SMase D are represented by GI numbers for each species. Class I isoforms are shown in bold. The number of sequences for each species (N) and total numbers are indicated (Σ). (PDF 121 kb)

## Abbreviations

SMase D: Sphingomyelinase D
