# Erratum: Adaptive evolution in the toxicity of a spider’s venom enzymes

**DOI:** 10.1186/s12862-016-0623-2

**Published:** 2016-03-07

**Authors:** Aurélio Pedroso, Sergio Russo Matioli, Mario Tyago Murakami, Giselle Pidde-Queiroz, Denise V. Tambourgi

**Affiliations:** Laboratório de Imunoquímica, Instituto Butantan, São Paulo, SP Brazil; Departamento de Genética e Biologia Evolutiva, Instituto de Biociências, Universidade de São Paulo, São Paulo, SP Brazil; Laboratório Nacional de Biociências, Centro Nacional de Pesquisa em Energia e Materiais, Campinas, SP Brazil

The original version of this article [1] was unfortunately published with a mistake. The figure legends for Figs. 8 and 9 were interchanged. The correct Figures and their associated legends are provided below:

**Fig. 8:**
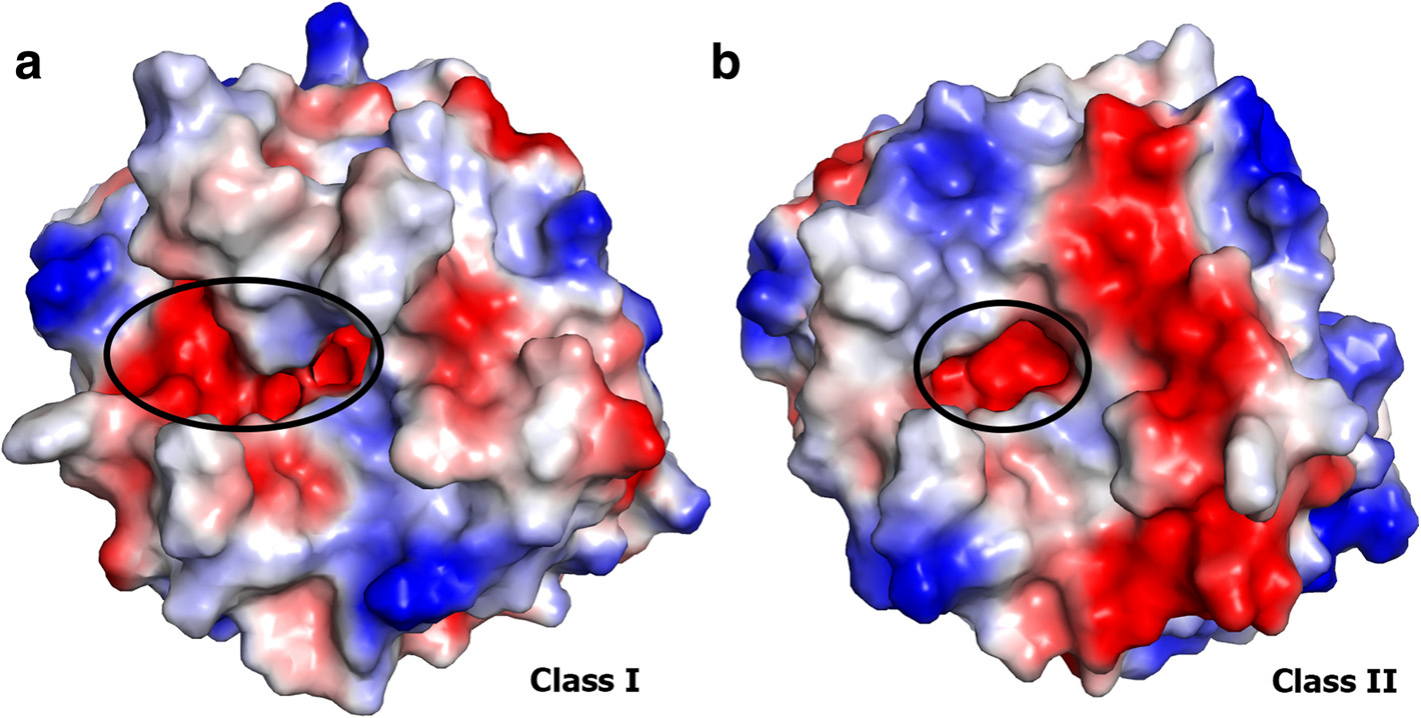
Electrostatic surface charge distribution of Class I and II SMases D highlighting the catalytic interface. The ellipses indicate the active-site pocket. **a** Class I; **b** Class II

**Fig. 9:**
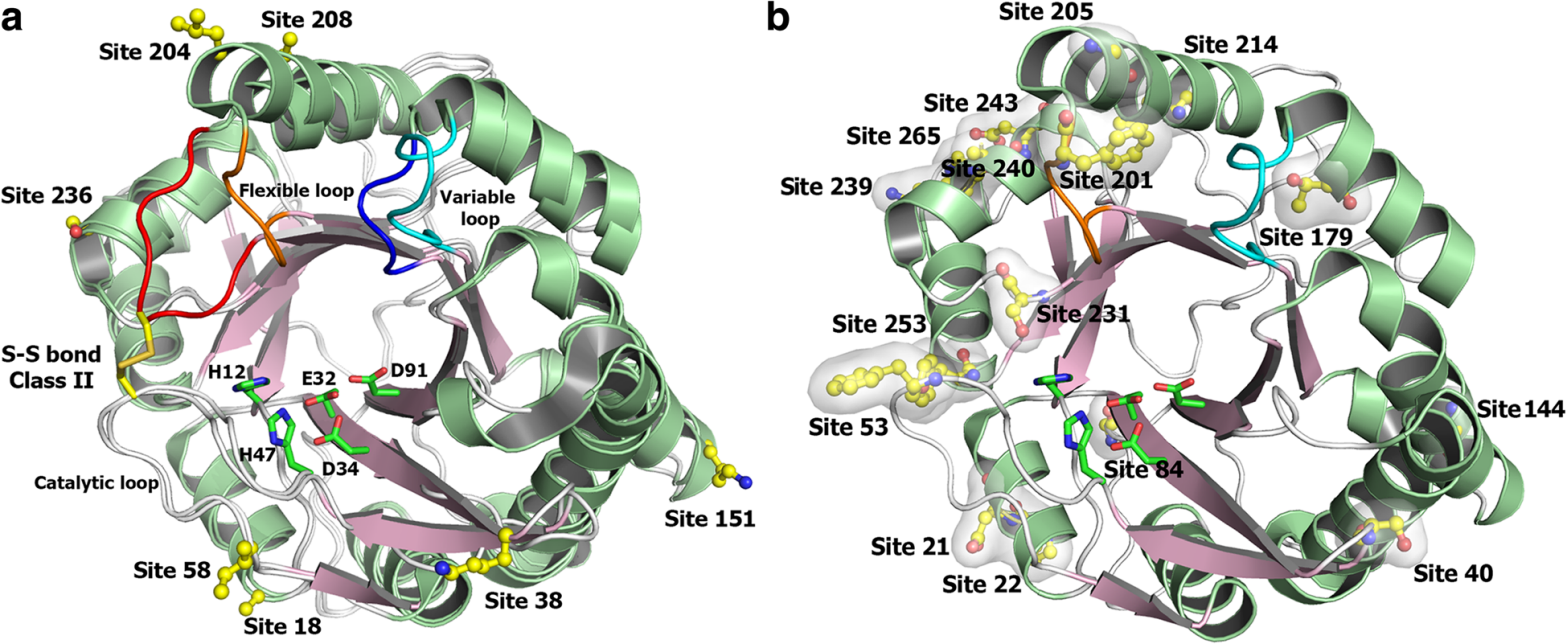
Structural interpretation of the positively selected sites. **a** Cartoon representation of the structural comparison between Class I and II SMases D. The fully conserved catalytic histidines (H12 and H47) and the three acidic residues (E32, D34 and D91) involved in the metal ion coordination are shown as sticks, with carbon atoms in *green*. The seven positively selected sites are shown as sticks and balls, with carbon atoms in *yellow*. The residues depicted in the positive sites correspond to those of SMase I from L. laeta, and the sequence numbering is also based on this molecule according to PDB entry 1XX1 (Murakami et al., 2005). The cartoon representation is coloured according to the secondary structure elements and the flexible loop F related to the second S-S bond found uniquely in Class II members (in *orange*, Class I; in *red*, Class II). The variable loop E is *cyan* and *blue* for Classes I and II, respectively. **b** Schematic representation of a Class I SMase D highlighting all of the positively selected sites for this class using the same colour pattern

Figure 8 Electrostatic surface charge distribution of Class I and II SMases D highlighting the catalytic interface. The ellipses indicate the active-site pocket. a Class I; b Class II.

Figure 9 Structural interpretation of the positively selected sites. a Cartoon representation of the structural comparison between Class I and II SMases D. The fully conserved catalytic histidines (H12 and H47) and the three acidic residues (E32, D34 and D91) involved in the metal ion coordination are shown as sticks, with carbon atoms in green. The seven positively selected sites are shown as sticks and balls, with carbon atoms in yellow. The residues depicted in the positive sites correspond to those of SMase I from L. laeta, and the sequence numbering is also based on this molecule according to PDB entry 1XX1 (Murakami et al., 2005). The cartoon representation is coloured according to the secondary structure elements and the flexible loop F related to the second S-S bond found uniquely in Class II members (in orange, Class I; in red, Class II). The variable loop E is cyan and blue for Classes I and II, respectively. b Schematic representation of a Class I SMase D highlighting all of the positively selected sites for this class using the same colour pattern.

## References

[1] Pedroso A, Matioli SR, Murakami MT, Pidde-Queiroz G, Tambourgi DV (2015). BMC Evol Biol.

