# Supplementary material for: Adaptive evolution in the toxicity of a spider’s venom enzymes
Source: BMC Evol Biol. 2015 Dec 21;15:290. doi: 10.1186/s12862-015-0561-4 (PMC4687385; doi:10.1186/s12862-015-0561-4)
Supplement: Additional file 1: Table S1. — DNA sequences from SMase D are represented by GI numbers for each species. Class I isoforms are shown in bold. The number of sequences for each species (N) and total numbers are indicated (Σ). (PDF 121 kb) [file 12862_2015_561_MOESM1_ESM.pdf]

Table S1. DNA sequences and amino acid sequences from SMase D are represented by GI numbers for each species. Class I isoforms are shown in bold. The number of sequences for each species (N) and total numbers are indicated (Σ).

TAXA	SMase D - DNA GI
***Loxosceles sp* (20 species)**	
**N**	**194**
L. hirsuta	224471908, 224471910, 224471912, 224471914, 224471916, 224471918, 224471920, 224471922, 224471924, 224471926, 224471928, 224471930, 224471932, 224471934, 224471936, 224471938, 224471940, 224471942, 224471944, 224471946, 224471966, 224471968, 224471970, 224471972, 224471974, 224472126, 224472128, 224472130, 224472132, 224472160, 224472162
L. intermedia	33348849, 41019462, 41019464, 77158010, 81248672, 81343345, 88660605, 88660607, 90192365, 90192367, 141452622, 156067377, 156067379, 156067381, 156067383, 156067385, 156067387, 156067389, 510937229
L. deserta	224471988, 224472026, 224472028, 224472030, 224472032, 224472034, 224472036, 224472038, 224472040, 224472042, 224472044, 224472046, 224472048, 224472050, 224472052, 224472054, 224472056, 224472058
L. arizonica	56694550, 56694552, 224471986, 224471990, 224471998, 224472000, 224472002, 224472018, 224472024, 224472114, 224472116, 224472142, 224472158
L. laeta	**27372515**, **27372517**, 27372519, **86795613**, **86795627**, **224472122**, **224472124**, 224472144, 224472146, 224472148, 224472150, 224472152, 224472156, 225008390, **310006474**, **310006476**, **LLAE0026C**, LLAE0066C, LLAE0072C, **LLAE0089C**, **LLAE0165C**, LLAE0175C, LLAE0193C, LLAE0194C, **LLAE0200C**, LLAE0223C, **LLAE0230C**, LLAE0245C, **LLAE0250C**, **LLAE0283C**, **LLAE0284C**
L. spadicea	224471948, 224471950, 224471952, 224471954, 224471956, 224471958, 224471960, 224471962, 224471964, 224471976, 224471978, 224471980, 224471982, 224472108

L. sabina	224471992, 224471994, 224471996, 224472060, 224472062, 224472064, 224472066,224472068, 224472070, 224472072, 224472074, 224472078, 225008386
L. spinulosa	224472180, 224472182, 224472184, 224472186, 224472188, 224472190, 224472192, 224472198, 224472282, 224472284, 224472286
L. apachea	224471984, 224472004, 224472006, 224472008, 224472010, 224472012, 224472014, 224472016, 224472020, 224472022, 224472112
L. variegata	224472080, 224472082, 224472086, 224472088, 224472090, 224472092, 224472094, 224472110, 225008388
L. rufescens	224472098, 224472100, 224472102, 224472104, 224472106, 224472140
L. amazonica	224472096, 224472134, 224472136, 224472138
L. reclusa	49458049, 49458051, 57792506
L. boneti	49458045, 49458047
L. sp. 4 GJB-2008	**224472118**, **224472120**
L. aff. spinulosa GJB-2008	224472194, 224472200
L. gaucho	**65336284**, 428230707
L. adelaida	353342964
L. similis	62275779
L. cf. spinulosa GJB-2008	224472178
***Sicarius sp* (5 species)**	
**N**	**46**
S. damarensis GJB-2008	224472208, 224472240, 224472242, 224472244, 224472246, 224472248, 224472250, 224472252, 224472254, 224472256, 224472258, 224472260, 224472262, 224472264, 224472266, 224472268, 224472270, 224472272, 224472274, 224472276, 224472278
S. peruensis	224472164, 224472166, 224472168, 224472170, 224472172, 224472174, 224472204, 224472210, 224472212, 224472214, 224472216, 224472218, 224472220, 224472222, 224472224, 224472226, 224472228, 224472232

S. patagonicus	224472202, 224472234, 224472236, 224472238
S. terrosus	224472176. 224472206
S. albospinosus	224472280
Corynebacterium sp	
**N**	**3**
C. pseudotuberculosis	289913, 289915, 512559
Arcanobacterium sp	
**N**	**2**
A. haemolyticum	289045, 257135781
Ixodes sp	
**N**	**3**
I. scapularis	89512120, 241641057, 241670277
Rhipicephalus sp	
**N**	**1**
R. pulchellus	427780840
**Σ**	**249**
